# Synchronous gastric cardia and antrum signet ring cell carcinomas: A rare case report highlighting diagnostic challenges and multidisciplinary management

**DOI:** 10.1097/MD.0000000000048009

**Published:** 2026-03-13

**Authors:** Chunmei Wang, Yu Su, Chunjing Jie, Tao Teng, Yan Zhang, Yin Chen, Peng Chen, Wenzhong Chen

**Affiliations:** a Digestive System Department, Tongren People’s Hospital, Tongren, China; b Digestive System Department, Red Flag Hospital affiliated to Mudanjiang Medical University, Mudanjiang, China.

**Keywords:** case report, signet ring cell carcinoma, synchronous primary gastric cancer

## Abstract

**Rationale:**

Gastric cancer is one of the most common malignant tumors in China. Gastric signet ring cell carcinoma (SRCC) belongs to highly malignant undifferentiated gastric cancer, originating from the epithelial cells of the gastric mucosa. Research has shown that the incidence of gastric cancer is gradually decreasing, but the incidence of gastric SRCC is gradually increasing. We report an extremely rare case of dual primary SRCC involving the gastric cardia and antrum. This case highlights the diagnostic challenges of synchronous SRCC, where a high level of suspicion, comprehensive endoscopic evaluation, and multidisciplinary management are crucial for achieving optimal treatment outcomes.

**Patient concerns:**

The 69-year-old female patient presented with poor appetite and significant weight loss, prompting her hospitalization. Following diagnostic evaluations, she was diagnosed with dual primary SRCC involving both the gastric cardia and antrum. Postsurgery, she experienced severe complications, including malnutrition, postoperative infections, and intestinal obstruction, significantly impairing her quality of life. Currently, she remains under regular chemotherapy (Tegio) and trastuzumab-targeted therapy.

**Diagnoses:**

The patient was finally diagnosed with double primary SRCC of the cardia and antrum of the stomach through gastroscopy and pathological tissue biopsy.

**Interventions:**

Laparoscopic radical total gastrectomy (October 28, 2024)

Adjuvant therapy: Tegio chemotherapy + trastuzumab (human epidermal growth factor receptor 2 [HER2] targeted)

**Outcomes:**

Complications such as malnutrition, postoperative infection, intestinal obstruction, as well as complications related to chemotherapy and targeted therapy, which had a serious impact on the quality of life.

**Lessons:**

Importance of clinical vigilance: Although this patient had no family history of gastric cancer, her nonspecific gastrointestinal symptoms ultimately led to the diagnosis of dual primary SRCC. This highlights the need for high suspicion of malignancy in elderly patients presenting with nonspecific symptoms.

Diagnostic value of multidisciplinary collaboration: The integration of endoscopic, pathological, imaging (computed tomography/magnetic resonance imaging), and immunohistochemical findings enabled accurate diagnosis of this rare case of dual primary gastric malignancy and identification of HER2 expression heterogeneity. The heterogeneity in HER2 expression (1+ vs 3+) demonstrates potential molecular differences between lesions in the same patient, emphasizing the necessity for comprehensive biopsy sampling.

## 1. Introduction

Gastric cancer is one of the common malignant tumors in the digestive system. Gastric signet ring cell carcinoma (SRCC) is a rare malignant tumor characterized by poor differentiation, high invasiveness, early metastasis, rapid progression, and poor prognosis.^[1]^ Synchronous primary gastric cancers (SPGCs) refers to 2 or more primary gastric tumors occurring simultaneously, separated by normal mucosa. SPGCs are not uncommon, with a reported incidence ranging from 1.1% to 15% among all gastric cancer patients.^[2]^ However, the majority of SPGCs are of the tubular adenocarcinoma type. Synchronous primary SRCCs are considerably less frequent, accounting for a smaller subset of SPGCs. More importantly, the simultaneous occurrence of SRCC at 2 distinct and distant anatomical sites – specifically, the gastric cardia and the antrum – represents an exceptionally rare clinical scenario. To the best of our knowledge, only a handful of similar cases have been sporadically reported in the literature.^[3]^ This rarity, combined with the significant diagnostic pitfalls and complex therapeutic decision-making it entails, underscores the value of reporting such cases in detail.

The cardia and antrum have different embryological origins, mucosal microenvironments, and cancer risk profiles, making such dual-site involvement by the same aggressive histological subtype both intriguing and clinically challenging.^[4]^ Due to the lack of characteristic clinical manifestations in the early stage, the diagnosis is somewhat challenging. The gold standard for early diagnosis of gastric SRCC is through endoscopic examination and biopsy of the lesion site for pathological analysis. The patient presented with poor appetite as the initial symptom at the gastroenterology clinic. Gastroscopy revealed lesions in the gastric cardia and antrum mucosa, and ultimately, histopathological examination diagnosed double primary SRCC of the gastric cardia and antrum. The diagnostic pitfalls, molecular heterogeneity (such as human epidermal growth factor receptor 2 [HER2] status), and implications for treatment strategies of these cases have not been fully explored. This report aims to emphasize the necessity of maintaining high vigilance and conducting comprehensive endoscopic evaluations in clinical practice through a case of this nature, and to explore the potential impact of its unique molecular characteristics on precise treatment decisions.^[5]^

## 2. Case report

**Figure 1.**
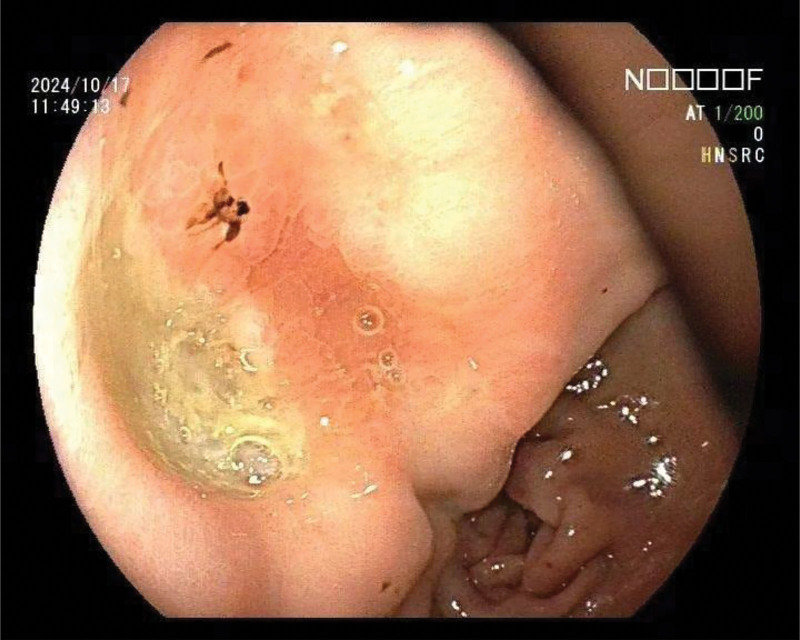
An irregular pitting ulcer was observed on the lesser curvature of the antrum of the stomach, approximately 1.2 cm in size. The base was covered with yellow white coating and filthy coating. The surrounding mucosa was edematous, and the sample was tough and prone to bleeding.

**Figure 2.**
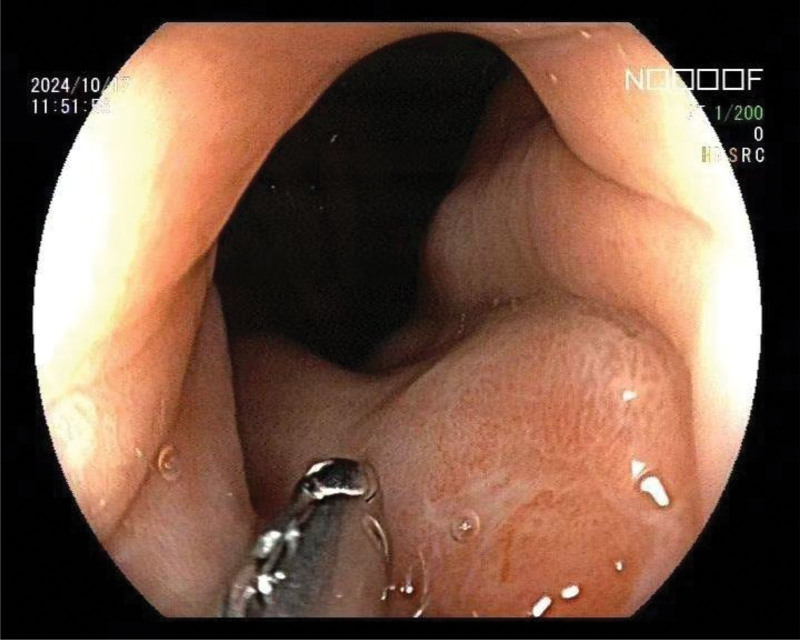
An irregular pitting ulcer was observed at the cardia, approximately 0.6 cm in size. The base was covered with yellowish-white coating. The surrounding mucosa was congested and edematous. The sample was tough and prone to bleeding.

**Figure 3.**
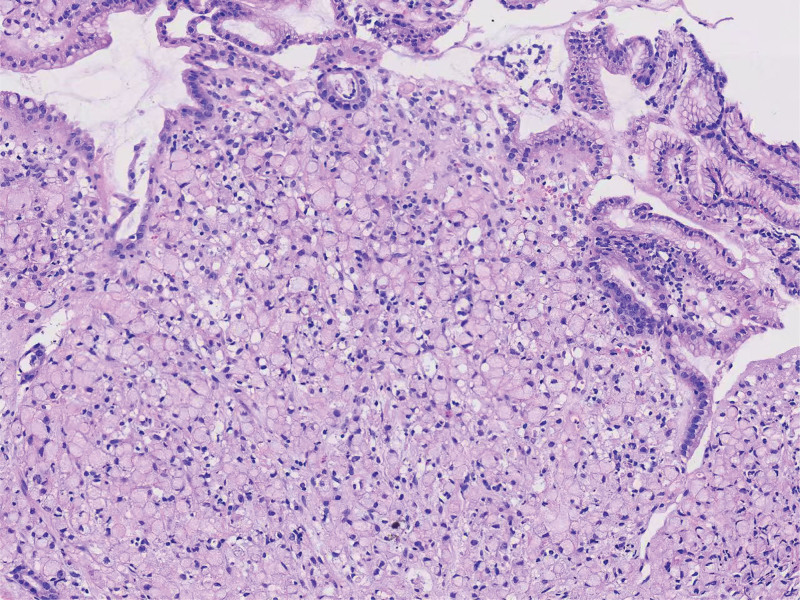
Signet ring cell carcinoma (small curvature of gastric antrum, gastric cardia).

**Figure 4.**
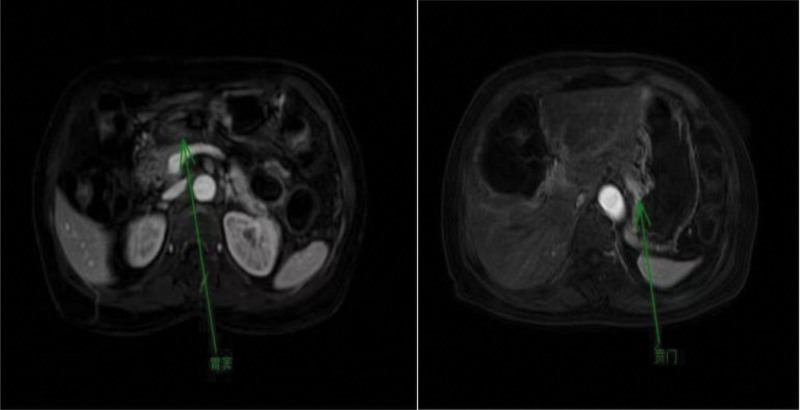
Plain scan of the upper abdomen + enhanced MRI indicated that there was local thickening and abnormal enhancement of the walls of the gastric cardia and antrum, without distant metastasis, which was consistent with the manifestations of gastric cancer (T2N1Mx). MRI = magnetic resonance imaging.

**Figure 5.**
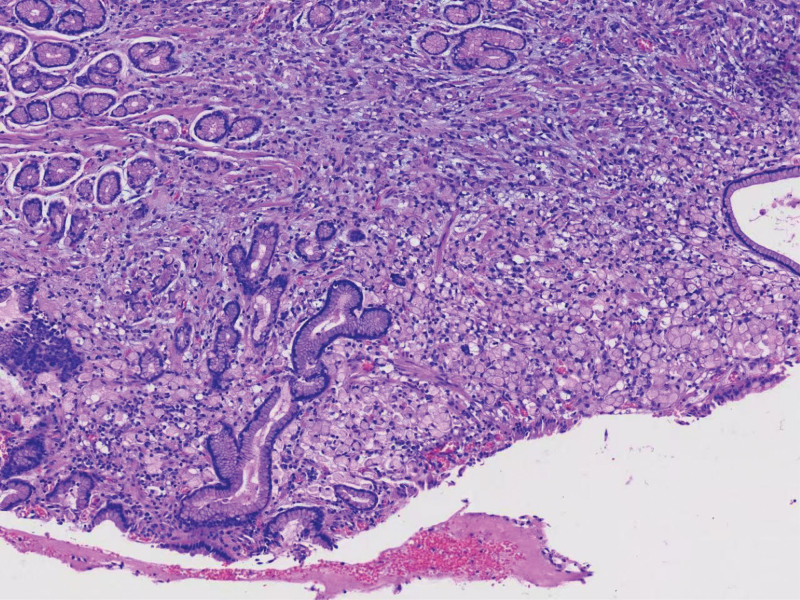
Postoperative pathological specimen (hematoxylin and eosin stain, low magnification). Microscopic examination confirms the presence of 2 independent signet ring cell carcinomas invading the muscularis propria. Lymph node metastasis was identified (pT2N3b).

The patient is a 69 year old female with a history of hypertension and no family history of HP infection or gastric cancer. The patient was admitted to the Gastroenterology Department of Tongren People’s Hospital in October 2024 due to “poor appetite with weight loss.” A comprehensive gastroscopy examination revealed an irregular concave ulcer on the lesser curvature of the gastric antrum, measuring approximately 1.2 cm in size. The base was covered with yellow daytime moss and dirty moss, and the surrounding mucosa was edematous. Four biopsy specimens were found to be tough and prone to bleeding (Fig. 1). An irregular depressed ulcer with a size of about 0.6 cm was observed in the cardia, with a yellow white coating covering the base and surrounding mucosa congested and edematous. Two tough and easily bleeding lesions were found on biopsy (Fig. 2). Pathological results suggest: SRCC (Fig. 3) in the lesser curvature of the gastric antrum and cardia. Based on the patient gastroscopy and pathological examination results, the diagnosis of primary SRCC in both the gastric cardia and antrum was confirmed, and the patient was admitted to the gastrointestinal surgery department for treatment. Upper abdominal plain scan + enhanced magnetic resonance imaging showed local thickening and abnormal enhancement of the gastric cardia and antrum walls, without distant metastasis, consistent with the appearance of gastric cancer (T2N1Mx) (Fig. 4). Laboratory examination: Digestive tract tumor markers indicate: carbohydrate antigen CA72-4 11.08 U/mL, ferritin 711.6 ng/mL. The result of the carbon-14 breath test was negative. After excluding surgical contraindications upon admission, a laparoscopic assisted radical total gastrectomy was performed on October 28, 2024. Pathological findings: microscopic examination of the resected total gastrectomy specimen confirmed the presence of 2 independent, synchronous primary SRCCs. The cardiac tumor measured 1.3 × 1.5 cm and invaded into the muscularis propria (pT2). The antral tumor measured 1.8 × 2.0 cm and also invaded into the muscularis propria (pT2). All surgical margins were free of carcinoma. Lymphovascular invasion was identified in both tumors, and metastasis was present in 20 out of 27 resected perigastric lymph nodes (pN2), the pathological stage was pT2N3b (Fig. 5). Immunohistochemical results: (cardiac tumor): cytokeratin (CK)-pan (+), CK20 (+), carcinoembryonic antigen (+), CK7 (+), Villin (+), mutL homolog 1 (+), postmeiotic segregation increased 2 (+), mutS homolog 2 (+), mutS homolog 6 (+), Ki-67 (10%+); (gastric antral tumor): CK-pan (+), carcinoembryonic antigen (+), CK7 (+), CK20 (+), Villin (+), Ki-67 (20%+). (tumors near the cardia): HER2 (1+) , HER2 (3+) tumor near the lesser curvature of the gastric antrum. The patient case was comprehensively reviewed at the institutional gastrointestinal oncology multidisciplinary team (MDT) meeting, which included medical oncologists, gastroenterologists, pathologists, and radiologists. Based on the imaging findings showing no distant metastasis (cT2N1M0) and the histopathological confirmation of 2 separate primary tumors, the MDT consensus was to proceed with curative-intent radical total gastrectomy with D2 lymphadenectomy. Furthermore, the pathologist highlighting of HER2 heterogeneity prompted a dedicated discussion among the medical oncologists. The decision was made to recommend adjuvant chemotherapy with Tegio chemotherapy in combination with trastuzumab, targeting the HER2 positive clone, while closely monitoring for potential differential response. During the follow-up process after surgery, the patient experienced complications such as malnutrition, postoperative infection, and intestinal obstruction, which had a serious impact on their quality of life.

## 3. Discussion

Gastric cancer is one of the most common malignant tumors in China, ranking 5th in the global cancer incidence rate and 4th in mortality.^[6]^ The prevalence of gastric cancer gradually declined, but the incidence of SRCC of the stomach gradually increased, accounting for 35% to 45% of the incidence rate of gastric adenocarcinoma.^[7]^ Gastric SRCC belongs to highly malignant undifferentiated gastric cancer, originating from the epithelial cells of the gastric mucosa. Its characteristic is that cancer cells produce a large amount of mucus, which squeezes the cells to 1 side, causing them to appear “signet ring” like under a microscope, hence the name.^[8]^ Regarding the location of the primary tumor, Shiotsuki et al,^[9]^ found that the primary location of undifferentiated early gastric cancer tumors is mostly in the middle 3rd of the stomach, while Wang et al.^[10]^ found that gastric SRCC and gastric adenocarcinoma are more likely to occur in the lower 3rd of the stomach, accounting for 69.6% and 70.8% respectively. SPGCs are not uncommon, with a reported incidence ranging from 1.1% to 15% among all gastric cancer patients. However, the majority of SPGCs are of the tubular adenocarcinoma type. Synchronous primary SRCCs are considerably less frequent, accounting for a smaller subset of SPGCs. Most reported synchronous gastric cancers (SGCs) consist of differentiated ad enocarcinoma or a mixed histology.^[11]^ Unlike the case reported by Smith et al^[11]^ where both lesions were HER2 negative, our case demonstrates striking molecular heterogeneity, a feature seldom documented in SGCs. This finding aligns with the growing recognition of spatial tumor heterogeneity as a key factor in precision medicine. Our case, therefore, contributes to the limited literature by emphasizing that SGCs can harbor distinct molecular profiles, a critical consideration that extends beyond mere diagnosis. It reinforces the need to manage each synchronous tumor as an independent entity for biomarker evaluation, which could significantly impact therapeutic strategy and clinical trial enrollment.

Early stage gastric SRCC lacks characteristic clinical manifestations, and the primary lesion can be detected through endoscopic examination. Histopathological examination of the lesion site is the gold standard for early diagnosis of gastric cancer. The commonly used endoscopic examination methods currently include white light endoscopy (WLE), narrowband imaging (an optical image enhancement technique that improves visualization of mucosal and vascular structures), magnifying endoscopy, endoscopic ultrasonography (used to assess the depth of tumor invasion and regional lymph nodes), etc. In our case, the diagnosis was confirmed through WLE combined with magnifying endoscopy and histopathological examination. According to the Japanese Gastric Cancer Association Guidelines for the treatment of gastric cancer, normal gastric mucosa appears uniformly pink or light red under WLE, with a smooth and glossy surface, visible regular gastric pits, clear vascular texture, uniform distribution, and no abnormal dilation or distortion; gastric SRCC may appear pale, grayish white, or red under WLE. Unlike the surrounding normal mucosa, the surface mucosa may be rough, uneven, and the structure of gastric pits may be disordered or disappear, forming ulcers, erosions, or elevations. The tissue is brittle and prone to bleeding. Under WLE, the patient presented with irregular concave ulcers on the gastric mucosa, with yellow and dirty moss attached to the base. The surrounding mucosa was congested and edematous, and the sample was tough and prone to bleeding.

Gastric SRCC can be treated with endoscopic therapy, surgical treatment, radiotherapy, chemotherapy, targeted immunotherapy, and other methods based on the depth of tumor infiltration, presence of lymph node metastasis, and distant metastasis. Early gastric cancer can be treated under endoscopy, and the main surgical treatments include endoscopic mucosal resection (EMR) and endoscopic submucosal dissection (ESD). In 2021, the 2nd edition of the Japanese Early Gastric Cancer ESD and EMR Guidelines stipulated that the absolute indication for EMR/ESD is non ulcerative cT1a differentiated cancer with a lesion length diameter of ≤2 cm. The absolute indications for ESD include: non ulcerative cT1a differentiated cancer with lesion length >2 cm; ulcer cT1a differentiated carcinoma with a length diameter of ≤3 cm; undifferentiated cT1a carcinoma without ulceration, with a length and diameter of ≤2 cm.^[12]^ The preferred treatment for advanced gastric SRCC is radical resection of gastric cancer, which involves removing at least 2/3 of the stomach and performing D2 lymph node dissection.^[12]^ Chemotherapy has made breakthrough progress in cancer treatment, and the comprehensive treatment of chemotherapy combined with surgery plays an important role in the treatment of gastric cancer. Adjuvant chemotherapy has also been proven to improve the survival rate of patients with advanced cancer after R0 resection by eradicating local and distant small lesions through chemotherapy. Due to the fact that most patients with gastric SRCC are already in the advanced stage when they seek medical attention, neoadjuvant chemotherapy can create surgical conditions for locally advanced patients. In this case, the patient had dual primary SRCC of the gastric cardia and antrum, both tumors in our case shared the same pathological T stage (pT2), indicating synchronous progression to the muscularis propria layer. This finding reinforces the concept that synchronous SRCCs may arise and progress within a similar timeframe under a common field effect or patient-specific risk factors. The identical depth also simplified the surgical staging, as both lesions unequivocally warranted radical total gastrectomy with D2 lymphadenectomy. Surgical radical gastrectomy was performed in combination with postoperative chemotherapy and targeted immunotherapy. During the follow-up process after surgery, related complications such as malnutrition, postoperative infection, and intestinal obstruction occurred, which seriously affected the quality of life.

HER2 is a member of the epidermal growth factor receptor family, a transmembrane glycoprotein with tyrosine kinase activity, which participates in the signal transduction regulation of cell proliferation, differentiation, and survival.^[13]^ In gastric SRCC, HER2 positivity usually refers to immunohistochemistry detection of 3+, or immunohistochemistry 2+ combined with fluorescence in situ hybridization confirmation of gene amplification (HER2/CEP17 ratio ≥ 2.0). The positive rate of HER2 in gastric SRCC varies, and this difference may be related to sample size, detection methods, interpretation standards, and tumor heterogeneity. HER2 is mainly overexpressed in gastric SRCC through mechanisms such as gene amplification, epigenetic regulation, and cross-talk of signaling pathways.^[13]^ HER2 overexpression is often associated with a high lymph node metastasis rate, advanced clinical stage, and other more aggressive clinical pathological features. HER2 interacts synergistically with the cluster of differentiation 44/C-X-C chemokine receptor type 4 axis to promote tumor invasion and metastasis.^[13]^ Currently, in terms of treatment, the main approach for gastric SRCC is radical surgical resection. With the continuous progress of medicine, tumor targeted therapy (targeted therapy) has become another new treatment modality. The main targeted drugs for HER2 are trastuzumab, pertuzumab, etc. HER2 positivity is an important predictive indicator for targeted therapy in gastric SRCC. The heterogeneity of HER2 expression observed in our case (1+ in the cardia vs 3+ in the antrum) presents a significant clinical challenge. This phenomenon, reported in 5% to 10% of gastric cancer cases, underscores the potential for sampling error in guiding targeted therapy decisions.^[14]^ The prognostic and predictive impact of intratumoral HER2 heterogeneity remains controversial. Some studies suggest that heterogeneous expression is associated with a lower response rate to trastuzumab compared to homogeneous strong positive (3+) cases.^[15]^ This highlights the critical importance of performing comprehensive biopsy sampling from multiple distinct tumor sites in patients with synchronous GC, as recommended by recent guidelines, to accurately assess HER2 status and identify patients who may truly benefit from anti-HER2 therapy.^[16]^ In our patient, the decision to administer trastuzumab was based on the presence of the HER2 positive (3+) component, acknowledging the potential for diminished efficacy due to heterogeneity

Research suggests that early stage gastric SRCC has a better prognosis than conventional gastric adenocarcinoma, while late stage gastric SRCC is a poor prognostic factor after radical resection of advanced gastric cancer.^[8]^ Franko et al,^[17]^ found that the prognosis of SRCC is related to its location, with colorectal SRCC having the worst prognosis, followed by the stomach. Tanaka et al,^[18]^ found that compared to nongastric SRCC, patients with early gastric SRCC have a longer postoperative survival time, with 5-year overall survival rates of 99.7% and 97.2%. Kim et al,^[19]^ found that the 5-year overall survival rate of gastric SRCC was 31.9%. Among non SRCC patients, the 5-year overall survival rates of highly, moderately, and poorly differentiated individuals were 45.1%, 38.4%, and 34.5%, respectively. The positive expression of HER2 is closely associated with a poorer prognosis for patients with gastric adenocarcinoma. Studies have shown that the 1-year, 2-year, and 3-year survival rates of HER2-positive patients with gastric adenocarcinoma (70.0%, 15.6%, and 0.0% respectively) are significantly lower than those of HER2-negative patients (96.1%, 73.3%, and 43.4% respectively).^[20]^ The median survival time of HER2-positive patients is only 15 months, while that of HER2-negative patients can reach 31 months.^[21]^ The positive expression of HER-2 is an important adverse prognostic indicator for gastric adenocarcinoma. Even in patients who have undergone radical surgery and adjuvant chemotherapy, the recurrence risk of HER2-positive patients may be higher and their survival time may be shorter.

The presentation of synchronous SRCCs in both the cardia and antrum offers several novel insights beyond merely documenting a rare entity. First, highlighting diagnostic pitfalls: the diffuse, infiltrative growth pattern of SRCC often leads to subtle or even normal-appearing mucosal findings on conventional endoscopy, especially in early stages. When this occurs at 2 remote sites, the likelihood of overlooking 1 lesion increases significantly, potentially leading to incomplete staging and suboptimal treatment planning. Our case underscores the imperative for a systematic and meticulous endoscopic examination of the entire stomach, including the use of advanced imaging techniques (such as magnifying endoscopy with narrowband imaging) and liberal biopsy practices in any area with even slight discoloration or rigidity, regardless of the presence of an obvious mass. Second, informing multidisciplinary management (MDT) strategies: the presence of dual SRCCs at opposing ends of the stomach presents a unique surgical dilemma. While total gastrectomy with D2 lymphadenectomy is the standard curative approach for diffuse-type or multi-focal gastric cancer, the decision becomes more complex when considering quality of life. This case vividly illustrates the critical role of a preoperative MDT discussion involving gastroenterologists, surgical oncologists, radiologists, and pathologists. The MDT must weigh the oncological necessity of complete resection against the functional consequences, potentially discussing the feasibility and risks of more limited resections (which are generally not recommended for SRCC) versus the unequivocal need for total gastrectomy in this setting. Our patient management pathway provides a concrete example of this complex decision-making process. Third, prompting questions on pathogenesis: the synchronous development of SRCC at 2 sites raises compelling questions about a field cancerization effect or a common underlying molecular driver. Future research could investigate whether such cases share specific genetic mutations (e.g., in cadherin 1 [E-cadherin], ras homolog family member A) or epigenetic alterations distinct from solitary SRCCs.^[21]^ Documenting these rare cases is the 1st step in accumulating data for such comparative analyses.

## 4. Conclusion

In conclusion, we present an exceedingly rare case of synchronous SRCCs exhibiting intratumoral HER2 heterogeneity. This case serves as a critical reminder that: synchronous gastric lesions should be meticulously evaluated and biopsied separately to avoid missing critical diagnostic and predictive information; the potential for biomarker heterogeneity must be considered in the MDT setting, as it directly influences the choice of targeted therapies and the interpretation of their efficacy; and a proactive, multidisciplinary approach is paramount not only for diagnosis and treatment planning but also for the long-term management of complex nutritional and functional sequelae following total gastrectomy. Future efforts should focus on establishing standardized protocols for the molecular evaluation of multiple synchronous tumors.

## Author contributions

**Conceptualization:** Yin Chen.

**Data curation:** Chunjing Jie, Tao Teng.

**Resources:** Yu Su, Yan Zhang, Peng Chen.

**Writing – original draft:** Chunmei Wang.

**Writing – review & editing:** Wenzhong Chen.
